# Recent advances in our understanding of NEC diagnosis, prognosis and surgical approach

**DOI:** 10.3389/fped.2023.1229850

**Published:** 2023-07-31

**Authors:** George S. Bethell, Nigel J. Hall

**Affiliations:** University Surgical Unit, Faculty of Medicine, University of Southampton, Southampton, United Kingdom

**Keywords:** necrotising enterocolitis, decision making, surgery, prognosis, prognostication

## Abstract

Necrotising enterocolitis (NEC) remains a devasting condition that has seen limited improvement in outcomes in recent years. The incidence of the disease is increasing as more extremely premature infants survive. NEC is responsible for 1 in 10 neonatal deaths and up to 61% of survivors have significant neurodevelopmental delay. The aim of this review is to highlight recent advances in diagnosis, prognosis and surgical approach in this condition. Many recent studies have reported novel methods of diagnosis of NEC with the aim of earlier and more accurate identification. These include imaging and machine learning techniques. Prognostication of NEC is particularly important to allow earlier escalation of therapy. Around 25% of infants with NEC will require surgery and recent data has shown that time from disease onset to surgery is greater in infants whose indication for surgery is failed medical management, rather than pneumoperitoneum. This indication was also associated with worse outcomes compared to pneumoperitoneum. Ongoing research has highlighted several new methods of disease prognostication which includes differentiating surgical from medical NEC. Finally, recent randomised controlled trials in surgical technique are discussed along with the implications of these for practice. Further, high quality research utilising multi-centre collaborations and high fidelity data from electronic patient records is needed to address the issues discussed and ultimately improve outcomes in NEC.

## Introduction

The incidence of necrotising enterocolitis (NEC) is increasing and outcomes in this condition have shown no improvement in recent years despite advancements in neonatal intensive care and improvements in outcome in a number of other conditions that effect premature infants (1). A recent systematic review and meta-analysis revealed that NEC is responsible for 1 in 10 neonatal deaths whilst 61% of survivors experience significant neurodevelopmental delay (2). Additionally, NEC is the most common cause of intestinal failure in children and parenteral nutrition is required in up to 9% of survivors of NEC at 1 year of age (3, 4). This has significant impact on children and families whilst creating a significant lifelong, financial burden on health and social care systems.

Research into the exact pathophysiology underlying NEC is ongoing and not fully understood however it is felt to be multifactorial involving a number of important molecular signalling mechanisms (5). Toll-like receptor 4 (TLR4) plays a crucial role in the development of NEC and is an immune receptor found in elevated frequency on enterocytes, intestinal stem cells and macrophages of prematurely born infants. TLR4 activation by microbial motifs, such as lipopolysaccharide, triggers a pro-inflammatory response which also induces apoptosis in enterocytes and inhibits enterocyte migration, contributing to intestinal injury (6, 7). TLR4 also supresses cell proliferation including those of the mucous barrier via the Wnt and Notch signalling pathways (8). Impairment of intestinal perfusion is another critical factor in the pathogenesis of NEC. Prematurely born infants intestinal vascular system demonstrates increased vasoconstriction leading to inadequate vasodilation in response to digestion (9). This leads to ischaemia and intestinal injury following feeding. Further vasoconstriction occurs due to reduced expression of nitric oxide synthase secondary to TLR4 activation. Downregulation of development of a premature infants microvasculature further contributes to ischaemia and necrosis in response to increased postnatal stresses such as feeding and bacterial colonisation moderated by the Vascular endothelial growth factor (VEGF) and VEGF receptor 2 (VEGFR2) pathways (10–12). Additionally, bacterial colonisation stimulates platelet-activating factor (PAF) leading to cell apoptosis. PAF has been shown to be increased in NEC and inhibition has been shown to have protective effects in animal studies (13). It is also clear that the immature gut immune system plays a significant role in development of NEC as lymphocytes and macrophages are pro-inflammatory compared to term infants (14).

Fortunately, there is ongoing research into all aspects of NEC with active research groups across the globe. Prevention of the disease has always been a key focus for researchers and recently there has been great interest in the use of probiotics. The microbiome is implicated in the pathogenesis of NEC (15). Studies have found a bloom of intestinal *Gammaproteobacteria* usually precedes NEC in many preterm infants and protective commensal bacteria such as *Bifidobacterium spp.* are less abundant in infants that develop NEC vs. controls (16, 17). Probiotics have been widely studied to alter the microbiome in infants at risk of NEC and prevent disease (18, 19). This work has culminated in a recent European Society of Paediatric Hepatology, Gastroenterology and Nutrition (ESPHGAN) specialist interest group recommending specific probiotic strains for the prevention of NEC (20). These strains are *L. rhamnosus GG* or a combination of *Bifidobacterium (B) infantis BB-02*, *B lactis BB-12*, and *Streptococcus thermophilus TH-4.* Numerous meta-analyses have been published reporting pooled data from trials of this intervention many of which have shown that probiotics are effective at reducing the incidence of NEC. However a recent Cochrane review of this area concludes that the certainty of evidence is low and the grade of recommendation is weak (21–23). Other techniques that are currently being evaluated for disease prevention include remote ischaemic conditioning. Remote ischaemic conditioning is a technique which has shown promise in animal models of NEC (24). It involves exposing an infant to periods of ischaemia, such as by torniquet of a limb, prior to developing disease which allows greater resilience to ischemia. Ischaemia is known to be a key element in the pathogenesis of NEC. Animal studies have shown that this method is particularly effective and significantly reduces the extent and severity of bowel injury compared to controls (24). At this stage human studies have not progressed beyond safety studies but further clinical research is in progress included a feasibility randomised controlled trial (25, 26). Human breast milk, either maternal or donor, has been shown to almost half the risk of NEC vs. formula feed in meta-analysis (27). The exact mechanisms for this are an area of ongoing research but *in-vitro* studies and animal models suggest that these mechanisms include epidermal growth factor (EGF) mediated inhibition of signalling via the innate immune receptor TLR4, human milk oligosaccharide (HMO) associated enhancement of intestinal perfusion and binding of intestinal bacteria by Immunoglobulin A (IgA) (28, 29). Whilst NEC continues to afflict preterm infants it is important that we can identify and treat NEC as quickly and effectively as possible. There have been recent advancements in diagnosis, management and prognostication which are discussed further in this article along with areas of future research.

## Diagnosis

Making an accurate and timely diagnosis of NEC continues to be a significant challenge (30). Other intestinal diseases such as septic ileus and focal intestinal perforation have similar clinical features including abdominal distention and global clinical deterioration. However, early and accurate diagnosis is essential to allow timely treatment for an appropriate duration. Moreover, good quality research in NEC is dependent on accurate differentiation of those with NEC from those with other conditions (31).

Criteria and scoring systems to diagnose NEC, and differentiate it from these other conditions, have been long established and include the Vermont-Oxford Network definition, Bell's criteria and a gestational-age specific scoring system (31–34). Data from a UK based collaboration were used to derive the gestational age specific case definition (31). Clinical and radiological features are assigned a score to give an overall score from 1 to 9. Whether the total score meets the criteria for NEC or not is determined by the gestational age of the infant. If an infant is less than 30 weeks gestational age then 2 points are required whereas 4 are required if the infant has a gestational age of 37 weeks or more. This was effective and using this approach achieved a sensitivity of 63.6% and specificity of 96.8% with a positive predictive value of 85.5%. More recently, machine learning has been employed to differentiate infants with NEC from those with other conditions. One study used these methods to differentiate NEC from focal intestinal perforation at a single centre with remarkable accuracy (35). A random forest model was able to differentiate these two conditions with a sensitivity of 96%, specificity of 96% and an area under the receiver operating characteristic (AUROC) of 0.98. The variables included in the model were pneumatosis intestinalis, pneumoperitoneum, corrected gestational age prior to surgery and gestational age at birth. Another study using machine learning in a modest cohort of infants found that definitions based on Bell were outperformed by novel artificial intelligence methods (36). The most effective model used the presence of apnoea, lethargy, Guaiac-positive gastrointestinal bleed, pneumatosis, gestation age, post-natal age at NEC onset, volume of feeding at NEC onset, disseminated intravascular coagulation and occult rectal bleeding to differentiate NEC from other conditions. Whether these techniques prove useful in clinical practice remains to be seen.

A metabolomics and proteomics approach to biomarker discovery for the diagnosis of NEC has attracted increased interest in recent years. This approach typically uses liquid chromatography-mass spectrometry to determine the presence of proteins and metabolites in fluids of cases and controls. Various different specimens have been investigated in infants with NEC which include stool, serum, urine, intestinal tissue and buccal swab samples (37). The challenges of this approach are the need for high quality samples, expertise in advanced biochemical techniques and access to specialist equipment. This hypothesis-free approach to biomarker discovery is particularly effective in experimental medicine and has had positive findings in a number of studies (38–42) along with some important reports of negative findings (43–45) mainly limited due to sample sizes. A study which shows particularly potential investigated a multi-centre cohort of infants with confirmed NEC, defined as meeting Bell's criteria, and controls who were healthy or had sepsis (40). Seven urine biomarkers were identified which delineated NEC from sepsis with an AUROC of 0.98. Genomics have also been investigated for the identification of NEC and several associations have been identified between genetic variants and disease (46). Individual genes that increase the risk of NEC include TLRR4, Single immunoglobulin and toll-interleukin 1 receptor (SIGIRR), Nucleotide binding oligomerization domain containing protein 2 (NOD2) and many others (46–49). Genome wide approaches have also been undertaken which found strongest association with a cluster of single nucleotide polymorphisms in chromosome 8 followed by chromosomes 14 and 11 (50). This recent and exciting approach may further uncover the pathogenesis of NEC whilst allowing better identification of those at risk of disease or with early disease.

Another method well known to neonatology but with little implementation with NEC is heart rate variability (51). A study of 245 infants, of which 32 had NEC, calculated heart rate variability using electrocardiogram (ECG) data combined with a panel of blood cytokine levels to diagnose NEC. Decreased heart rate variability was associated with a diagnosis of NEC although the numbers studied were low and the clinical utility of this from this current study is limited (52). Given the ability for heart rate variability to improve detection and outcomes in neonatal sepsis this is certainly an area for further exploration (53, 54).

Abdominal ultrasonography (US) has also gained interest in recent years with many studies exploring the utility of this modality in NEC diagnosis. A recent systematic review and meta-analysis summarised 6 studies which included 462 children evaluating the use of US to diagnose NEC (55). A number of US signs were taken individually including portal venous gas, free air, pneumatosis intestinalis, bowel wall thinning and simple ascites. All these signs were found to have a pooled specificity of between 91% and 99%. The pooled sensitivity however was much lower and between 22% and 48% showing that US is a good modality for excluding NEC however less effective at diagnosing it. The important caveat is that these data are based on individual signs rather than a combined overall impression by an experience paediatric sonographer.

These recent studies all show promise for earlier diagnosis of disease however there are some limitations to overcome prior to incorporation into clinical practice. The majority of which are related to incorporation of these methods into current electronic patient records and real-time monitoring systems. Even the most accurate method of prediction, developed from sophisticated statistical or machine learning methods, requires implementation into bedside systems so that these earlier diagnoses are brought to the attention of clinicians in real time. It is hoped that earlier treatment, including administration of antibiotics, cessation of enteral feeding, advanced monitoring and multi-organ support will limit disease progression. This assumption is yet to be confirmed.

## Prognostication

Prognostication in NEC is being recognized as increasingly important. A quarter of babies with NEC undergo acute surgery due to bowel perforation, clinical deterioration with maximal medical therapy or failure to recover (56). After the initial acute episode there is a further cohort of infants that develop stricture formation and may require surgery for this (57). It is anticipated that accurate identification of those with severe NEC early in the disease course will allow earlier surgical intervention. Recent observational data suggest that those infants with NEC that have the longest time from diagnosis to surgery have the worst outcomes. In a secondary analysis of a population-based study infants were grouped depending on indication for surgery as determined by the operating surgeon. Those that underwent surgery on the basis that they were deemed to have failed medical therapy had surgery (adjusted) 30 h later than those with bowel perforation. This same group of infants were 4.5 times as likely to require parenteral nutrition or have died by 28 days following surgery (56). Requirement for parenteral nutrition at 28 days post surgery has previously been shown to be associated with mortality at 1 year follow-up (3). These data suggest that earlier identification of need for surgery in NEC, accompanied by earlier surgery has the potential to improve outcome. These data are however limited by their observational nature and lack of consistent definition regarding whether surgery is indicated or not. For example some infants that underwent surgery may have improved without intervention although reassuringly no intervention at laparotomy was only required in 3% of this cohort (56). Additionally, as many as 20% of infants with NEC die of the disease prior to ever undergoing surgery although it is impossible to know whether surgery would have changed this outcome (58). Moreover, in 1 in 20 that do undergo surgery the extent of necrosis is so great that survival is not possible suggesting that earlier intervention would be of benefit (59). Identification of this group of babies earlier may be key to improving survival and outcomes.

Earlier identification of need for escalation of medical treatment and requirement for surgery are also likely to improve longer term outcomes. Poor neurodevelopmental outcomes in survivors of NEC is thought to be secondary to reduced cerebral perfusion and exposure of the developing brain to prolonged systemic inflammation which occurs in severe NEC (2, 60, 61). Mouse studies have shown that activation of microglial cells in the brain promote cognitive impairment secondary to production of Toll-like receptor 4 endogenous ligands by inflamed intestine (62). Additionally, in this study it was possible to prevent cognitive impairment with administration of microglia-targeting antioxidants (62). This suggests that medical therapies may be key to unlocking better long term outcomes in NEC however human study of this is required. In the meantime, it is hypothesised that earlier removal of diseased intestine reduces cerebral exposure to these harmful substances and hence reduces cerebral tissue damage with the caveat that it is unknown as to whether surgery itself detrimentally impacts cerebral perfusion due to physiological stress and increased exposure to anaesthetic agents. Nevertheless, to test this hypothesis we require accurate and early identification of intestinal necrosis, preferably in a non-invasive manner. Many methods have been derived to differentiate those with medical NEC from those that require surgery, known as surgical NEC. These include various biochemical biomarkers in blood plasma, urine and stool that are not yet readily clinically available (63–68) along with novel machine learning approaches (39). Additionally, conventional biochemical biomarkers that are readily clinically available have also been investigated (69–71) along with the use of scoring systems (72, 73). Novel methods requiring specialised equipment in the form of Near-Infrared Spectroscopy (NIRS) (74) and heart rate variability (75) have both shown promise in small studies. Finally, imaging methods have been extensively explored for this purpose (55, 76).

### Biomarkers

There have been many promising studies published in recent years. Firstly, authors of a retrospective UK based study including 191 infants with non-perforated NEC hypothesised that a serum c-reactive protein (CRP) to serum albumin ratio could predict surgery and also mortality (77). It was found that a CRP to albumin ratio of more than or equal to three on day two of NEC diagnosis was most effective at predicting surgical intervention with an AUROC of 0.71 and was slightly less effective at predicting mortality (AUROC = 0.66). This study addresses the group of most interest which is those with non-perforated disease as this is where decision making is most challenging (56) and the results of prospective use of this method are much awaited.

Another recent study focussing on readily available clinical data retrospectively investigated the ability of the coagulation profile, 12 h after disease diagnosis to predict surgical intervention (78). In 114 infants, where the rate of surgical intervention was 40%, the presence of coagulopathy was defined as a platelet count less than 100 × 10^9^/L or an activated partial thrombo-plastin time greater than 45.4 s or a prothrombin time international normalized ratio greater than 1.3. It was found that the presence of coagulopathy at this timepoint was predictive of surgical intervention with AUROC of 0.869 and a specificity of 91.2% which outperformed individual tests from the coagulation profile within the same study. These results are exciting but again require prospective evaluation and consideration of how the effectiveness of this method changes depending on point of definite diagnosis. It is relatively easy to decide retrospectively the point in which NEC was diagnosed but more challenging in real world settings.

A collaborative study involving multiple institutions in the Netherlands investigating biomarkers for NEC detection and late-onset sepsis separately looked at a cohort of infants in this study with medical NEC and compared these, to those that underwent surgical intervention for NEC (79, 80). Rather than explore the ability of patient characteristics, clinical features or laboratory results to predict those who underwent surgery and those who didn't, associations between these two groups were sought. Multivariable regression was used to adjust for confounding and it was found that surgical NEC was associated with lower gestational age, no maternal corticosteroid administration, earlier onset of NEC, lower serum bicarbonate (prior to disease onset) and a hemodynamically significant patent ductus arteriosus for which ibuprofen was administered. These results are interesting and can certainly be incorporated into further work looking at better ways to prognosticate in this condition but arguably cannot be implemented in the neonatal intensive care unit yet. Additionally, it may be challenging to convince clinicians of the importance of a factor such as maternal corticosteroid administration. Despite showing statistical significance it is very unlikely that neonatologists or surgeons consider this in practice.

### Imaging

Abdominal US has been investigated as a radiological method of determining surgical from medical NEC. A systematic review by *Cuna* et al. included 11 studies of which 2 were prospective (55). It was found that there were several features that were associated with surgery or death of which a focal fluid collection, complex ascites and absent peristalsis had the highest odds ratios. The authors conclude that further work is needed to assess whether using this technique improves outcome and when it should be undertaken. A practical limitation of US is that it requires a sonographer with experience of using US in NEC and results in a snapshot of abdominal signs at the time of study. As this is not routine practice it can be difficult and slow to arrange in reality (81).

An alternative radiological method that has for the first time been investigated to differentiate medical from surgical NEC is computed tomography (CT) imaging. Abdominal CT imaging is frequently used in adults to accurately identify ischaemic or necrotic bowel in conditions such as small bowel obstruction or mesenteric ischaemia. It is highly effective in these settings but is rarely undertaken for any indication in premature infants. However, in a study of 34 infants with clinical and radiological features of NEC, 21 participants underwent abdominal dual energy CT scan (76). The mean weight of infants at time of imaging was just over 1.3 kg with a standard deviation of +/− 0.53 kg. Bowel ischaemia was identified in 9 infants whom subsequently had a laparotomy where ischaemic bowel was found and confirmed histologically. The sensitivity, specificity, positive predictive value and negative predictive values in this study were all 100%. This highly effective approach has similar limitations to US, it requires a highly skilled paediatric radiologist to interpret findings and provides a snapshot of intra-abdominal signs at the time the scan was undertaken. The challenge of moving a critically unwell infant to the CT scanner may also contraindicate this method in real world settings. More detail regarding logistics and timing of these studies is needed to further inform clinicians about the true feasibility of this method.

### Summary

The studies discussed here clearly highlight the wealth of research currently being undertaken in this area which has significant importance to all stakeholders. Each method has its advantages but most need further investigation or development before they can be implemented into routine clinical practice. Moreover, incorporation of these, non-radiological, methods into electronic real-time monitoring systems is an essential prerequisite. Most studies into this problem are from single centres and hence only include a handful of patients with NEC. This is a problem for most studies, but particularly those using machine learning where large numbers of participants are required to effectively train models. Multi-centre collaboration is needed to increase the effectiveness of these whilst also ensuring they remain generalisable to populations beyond single neonatal units. These studies are harder to undertake, requiring ethical approval, data sharing agreements and restructuring of data to allow combination into one dataset but these challenges are not insurmountable.

## Surgical approach

The principle of surgery for NEC is to reduce contamination and sepsis by control of bowel perforation and resection of non-viable intestine (82, 83). It is also essential to reduce physiological burden on the infant as much as possible by limiting surgical time, ensuring adequate systemic perfusion and avoiding hypothermia which can lead to life threatening coagulopathy (84). Many surgical approaches exist including peritoneal drainage, laparotomy with or without bowel resection, enterostomy formation or primary anastomosis and temporary laparostomy formation (59). The choice of procedure is dependent on extent of disease, surgeon preference and physiological status of the infant, with a significant lack of high quality evidence to guide clinical decisions.

One option for surgical intervention in perforated NEC is insertion of an intra-peritoneal drain rather than undertake a laparotomy. This is less invasive, quicker and reduces the physiological burden on the infant. Randomised controlled trials have explored whether this approach is advantageous in NEC but have shown no difference in outcomes using peritoneal drainage vs. laparotomy (85, 86). However, the most recent trial exploring this question included those with both NEC and focal intestinal perforation and recorded outcomes to 2 years following intervention (87). It was found that rates of death and neurodevelopmental impairment were similar between both treatment modalities when both diseases are pooled together. However, planned subgroup analysis revealed that for infants with a presumed diagnosis of NEC, death or neurodevelopmental impairment was seen more frequently in those with an initial peritoneal drain (85%) than with laparotomy (69%). This difference equates to a 97% likelihood of reducing mortality or neurodevelopmental impairment at 18–22 months corrected gestational age with initial laparotomy in NEC. This is likely due to NEC causing extensive bowel necrosis requiring resection. If necrotic bowel is removed then systemic inflammatory response will be reduced.

Protocolisation of all areas of medicine has become increasingly popular. This approach allows standardisation and allows evidence based practice even in infrequently encountered conditions such as NEC. A recent multi-centre study from the United States has described their protocol for determining surgical approach in NEC or focal intestinal perforation and the outcomes associated with this (88). The authors report that peritoneal drainage or laparotomy is undertaken in those determined to have surgical NEC or focal intestinal perforation depending on weight, age and abdominal radiograph findings. If an infant weighed less than 750 grams, was less than or equal to 14 days old and had either a normal or gasless or pneumoperitoneum on radiograph they underwent peritoneal drainage. All others underwent laparotomy. Those with a drain were monitored closely with planned drain removal at 7 days, but laparotomy if deterioration or no improvement occurred. This protocol meant that only peritoneal drainage, without subsequent laparotomy, was used in 27% of children after implementation compared to 13% prior to implementation. Despite this, no improvement was observed in survival after implementation of the protocol and further reports of this are awaited.

The concept of damage control surgery in NEC was first reported in 2004 (89). More recently a more detailed description of this technique and the potential benefits has been reported (84). In Birmingham Children's Hospital (Birmingham, UK), neonates who were severely unwell with presumed abdominal pathology underwent laparotomy on the paediatric intensive care unit. This took place as soon as possible with ongoing resuscitation during surgery. The aim of the initial procedure was to excise obviously dead or perforated bowel and then leave a laparostomy for planned relook surgery 48 h later. Surgery was undertaken as promptly as possible to limit physiological deterioration with a median operative time of 38 min. Only 13% of those with NEC required an enterostomy at relook laparotomy as most underwent delayed anastomosis. Mortality was seen in 18% of those with NEC at 28 days which is lower than most previously reported series (2). This technique requires coordination between all team members include transfusion laboratories to allow this approach. Other UK centres are currently developing similar approaches for selected infants.

These studies highlight recent developments in regards to surgery for NEC however it is challenging to robustly compare different surgical procedures in such a heterogenous population where there are no set standards for deciding whether surgery is indicated, or not. Providing the principles of surgery for NEC are met then it is likely that all surgical options will be comparable depending on operative findings in these challenging procedures.

## Further areas for research

Fortunately, there is plenty of interest in ongoing research of all aspects of NEC as highlighted throughout this review. Multi-centre collaboration is essential in this infrequently encountered condition, particularly when studying sub-groups such as those with surgical NEC. Important areas for further work include earlier detection of disease and better prognostication which includes earlier identification of need for care escalation and requirement for surgery. These questions will be easier to address in the age of technology driven healthcare, electronic patient records and advanced statistical techniques including machine learning. The ability of studies to address these issues is dependent of quality of data collection and it is more important, now than ever, to ensure that those with NEC are correctly identified in datasets. Those with other disease such as focal intestinal perforation should be correctly labelled as such. With coordinated efforts from all clinicians and researchers interested in this devasting condition it is hoped that the currently poor outcomes will improve for generations of future NEC sufferers and their families.
